# Position Estimation of Vehicle Based on Magnetic Marker: Time-Division Position Correction

**DOI:** 10.3390/s21248274

**Published:** 2021-12-10

**Authors:** Yeun Sub Byun, Rag Gyo Jeong

**Affiliations:** Korea Railroad Research Institute, 176, Cheoldobangmulgwan-ro, Uiwang 16105, Gyeonggi-do, Korea; rgjeong@krri.re.kr

**Keywords:** automatic driving, error estimation, position detection, guidance control, magnetic marker, magnetic sensing ruler, peak detection, autonomous vehicle, heading angle

## Abstract

During the automatic driving of a vehicle, the vehicle’s positional information is important for vehicle driving control. If fixed-point land markers such as magnetic markers are used, the vehicle’s current position error can be calculated only when a marker is detected while driving, and this error can be used to correct the estimation position. Therefore, correction information is used irregularly and intermittently according to the installation intervals of the magnetic markers and the driving speed. If the detected errors are corrected all at once using the position correction method, discontinuity of the position information can occur. This problem causes instability in the vehicle’s route guidance control because the position error fluctuates as the vehicle’s speed increases. We devised a time-division position correction method that calculates the error using the absolute position of the magnetic marker, which is estimated when the magnetic marker is detected, along with the absolute position information from the magnetic marker database. Instead of correcting the error at once when the position and heading errors are corrected, the correction is performed by dividing the errors multiple times until the next magnetic marker is detected. This prevents sudden discontinuity of the vehicle position information, and the calculated correction amount is used without loss to obtain stable and continuous position information. We conducted driving tests to compare the performances of the proposed algorithm and conventional methods. We compared the continuity of the position information and the mean error and confirmed the superiority of the proposed method in terms of these aspects.

## 1. Introduction

This study proposes a time-division (space-division) position correction method for use in automatic driving vehicles to minimize the position information discontinuity and the correction information loss that can occur in the classic position estimation method using magnetic markers as land marker methods.

Position estimation technology for automatic driving has been used in a variety of ways, not only for autonomous vehicles but also for automatic logistics transportation systems in places such as factories and ports. Using this technique, various devices and technologies are combined, such as global positioning system (GPS) technologies [1,2,3,4,5,6,7], camera-based image processing technologies [8,9,10], and laser-scanner-related technologies [11,12,13,14]. Among these various systems, land marker methods of positioning technology are used mainly in areas with limited operating range, such as factories and ports. Position detection systems based on a transponder method and a magnetic marker sensing method, one of the more popular land-marker-based positioning methods, have relatively low installation and maintenance costs and are less affected than other methods by changes in indoor and outdoor environments [15,16,17,18,19,20,21,22]. Furthermore, 2getthere [23] is a company that has applied this method to small passenger transport vehicles in a pilot operation in Masdar City, Abu Dhabi, United Arab Emirates.

Previous researchers [24] proposed a position estimation method using magnetic marker sensing and GPS. When such magnetic markers are used, the vehicle uses a method for estimating the absolute position of the vehicle while driving, whereby the magnetic detection sensor mounted on the vehicle detects magnetic markers buried in roads [16,17,18]. In this method, the position error can be measured at each magnetic marker position based on which error of the calculated position is corrected. Here, a Kalman filter and various sensors, including the vehicle’s speed sensor and rotational angular velocity, are combined to calculate the real-time position at each control cycle [16]. When the magnetic signal is collected just once in each control cycle and applied to the position estimation in the conventional magnetic detection signal processing method, there are limitations to the maximum driving speed of the vehicle because of the sampling time problem related to the magnetic signals. In other words, if the vehicle speed is too high compared to the signal collection cycle, the sampling of the signals may be insufficient to find the peak of the magnetic signals when passing over the magnetic marker. In this method, the position error is calculated whenever a magnetic marker is detected, meaning that when correcting the errors all at once in the corresponding cycle, if the correction error is large the corrected position information in the pertinent cycle may be discontinuous from the previous position. If a value smaller than the measured correction value is applied, the discontinuity can be mitigated, although this causes a loss of correction information. In conventional methods, if the discontinuity is mitigated by adjusting the setting of the Kalman filter coefficient value, a position delay occurs, causing a larger rotational radius compared to the actual driving position when driving on a curve. This error correction problem has a significant impact on the safety of the rotational driving control of the moving object. In other words, the longitudinal and lateral position estimation errors increase, reducing the driving precision and stability in the route guidance control using this method.

In this study, we propose methods to solve the two major problems presented:

- First, to improve the vehicle-speed-related magnetic signal processing problem, a signal processor is implemented to process magnetic signal detection at a fast cycle (1 ms), separate from the low vehicle control cycle (50 ms);
- A position correction method is proposed to reduce the loss of correction values and improve the position discontinuity that results from correcting errors. A time-division error correction method based on the moving distance is proposed for the errors measured when the magnetic markers are detected.

Based on this, we increased the speed band for position estimation by increasing the magnetic signal sampling cycle. The signal detection range of the magnetic marker by the magnetic sensor confirmed through the test is about 30 cm. This range is sufficient to detect the center of the magnetic signal if at least eight samples are obtained at 3 cm intervals of the signal, including the magnetic field center. Therefore, considering the travel distance of 3 cm in 1 ms (the magnetic signal sampling time), the theoretical maximum utilization speed of the developed MSR is 100 km/h. Furthermore, we used the measured correction amount while mitigating the conversion impact caused by sudden position corrections. Thus, the driving stability and ride comfort can be improved based on driving control using smooth and continuous position information when driving the vehicle.

The remainder of this paper is organized as follows. Section 2 describes the structures of the magnet-marker-based driving control system and the developed magnetic sensing ruler (MSR). Next, Section 3 suggests a method for estimating the magnet marker peaks through the detection of their magnetic signals, as measured while driving. Section 4 describes the absolute position estimation method of a vehicle, which combines the vehicle’s sensor information and the magnet marker position information measured using the MSR. Section 5 presents comparative test results with conventional methods to verify the proposed position correction method’s effectiveness and validity. Section 6 provides the conclusion.

## 2. Magnetic-Marker-Based Guidance System

**Figure 1:**
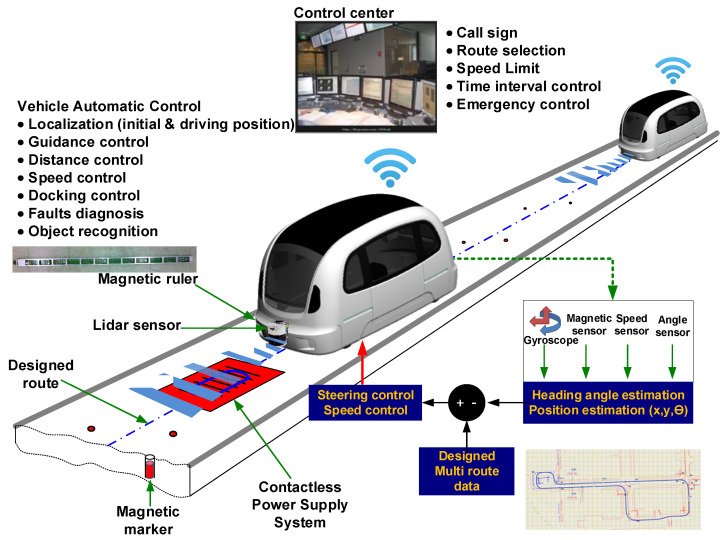
Schematic of the automatic guidance system based on magnetic markers.

To measure the vehicle’s position while driving in the vehicle’s automatic driving control system, an MSR was installed and cylindrical magnet markers (15 mm in diameter and 30 mm in length) were buried along the driving track. The MSR was installed parallel to the axle at the bottom of the vehicle at a height of approximately 12 cm. The vehicle’s host computer combines the information obtained from the gyroscope, steering angle sensor, and MSR installed on the vehicle to calculate the vehicle’s moving distance, direction, and position in the unit sample. Based on this information, a variety of control information required for the vehicle’s automatic driving was calculated. The developed vehicle was operated and controlled by a distant control center, as shown in Figure 1.

The magnetic signals of the magnet marker have a Gaussian function shape centered on the magnet marker. As the center of the buried magnet marker is approached, the strength of the magnet signals increases, and the highest strength of the magnetic signals is found at the center of the magnet marker. To measure the absolute position of the vehicle in real-time while driving, the relative longitudinal and lateral distances from the magnetic marker’s center to the MSR are calculated by analyzing the magnetic signals of the magnetic marker buried in the road. This information is used to calculate the absolute position of the vehicle based on the absolute position database information of the detected magnet marker. A variety of signal and control information for the vehicle is collected and determined in 50 ms cycles.

### Magnet Measurement System

#### Configuration of the Magnetic Sensing Rules

**Figure 2:**
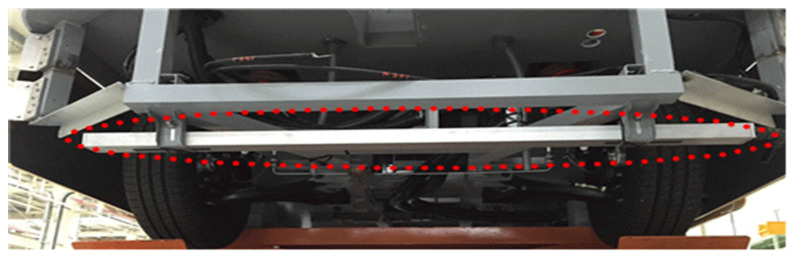
Installation of the magnetic sensing ruler (MSR).

**Figure 3:**
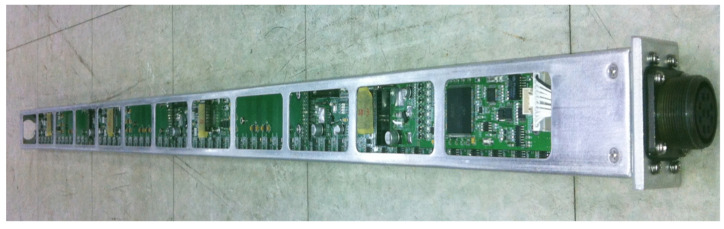
Magnetic sensing ruler (MSR).

The MSR is mounted at the bottom of the vehicle, as shown in Figure 2. It consists of 60 magnetic sensors arranged in 2 cm intervals in a line with an effective sensor measurement length of 120 cm, as shown in Figure 3. The magnetic sensors in the MSR collect magnetic information in 1 ms intervals and send the collected data to the signal analysis process every 50 ms via Ethernet. The MSR receives the reference synchronization number from the vehicle’s signal processing device every 50 ms and uses it as the reference detection time index of the collected magnetic signal. The signal processing device synchronizes the in-vehicle processing information using the time index included in the magnetic signal transmitted by the MSR and the current time index of the vehicle signal processing device.

## 3. Magnetic Marker Detection and Signal Processing

### 3.1. Magnetic Marker Position Detection

**Figure 4:**
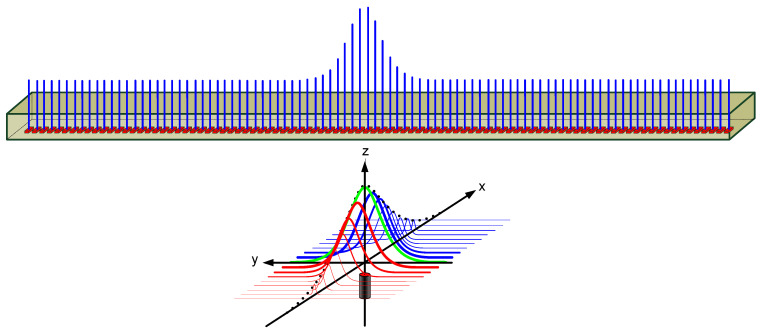
Magnetic marker and magnetic sensing ruler.

As shown in Figure 4, Hall effect sensors were arranged in certain intervals on the MSR installed at the bottom of the vehicle to measure magnetic signals at each sampling time. Because magnetic signals are collected as one-dimensional (1D) data according to the sampling cycle in a time sequence, the longitudinal center position of a magnet marker in the proceeding direction can always be checked after the MSR has passed the marker. Therefore, time and spatial delays inevitably occur in the recognition time-point of the center of the magnet marker because the collected 1D data must be arranged to construct and analyze 2D spatial data. The following procedure is performed on the collected time-series magnetic signal array data to check the sensing position of the magnet marker on the spatial coordinate system.

#### 3.1.1. Resampling

The values that must be checked from the collected magnetic signals are the relative longitudinal and lateral distances from the magnetic sensor axis to the center of the magnet marker at the reference time (50 ms). However, the data sent by the MSR are array information of the time-series magnetic signal data collected every 50 ms, measured in 1 ms cycles. The obtained signals have a normalized magnetic signal shape that does not change in the lateral direction (i.e., along the MSR’s length), although the magnetic signal shape on the time axis changes according to the vehicle speed in the longitudinal (i.e., proceeding) direction, which can cause difficulties in the process of finding the peak of the magnetic signals. Therefore, we performed a process of resampling the collected time-series data of the magnetic signals into spatial coordinate data. By doing so, we obtained spatially consistent magnetic signal distribution data regardless of the vehicle’s speed and effectively found the peak (the magnet marker’s center) position of magnetic signals on the magnetic sensor axis at the reference time from the data distribution.

**Figure 5:**
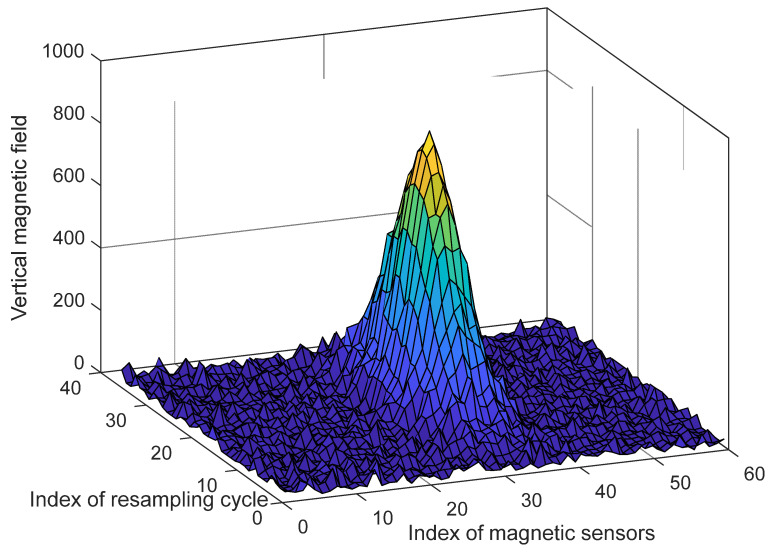
Magnetic field signal collected by the magnetic sensing ruler.

If there is no data resampling process, the size of the data collected on the time axis will increase when the vehicle passes the magnet marker at a low speed compared to when it passes at a high speed. Furthermore, if the MSR is above the magnet marker when the vehicle stops, the amount of data to be analyzed will increase dramatically, and a significant amount of computational time will be required to process the data. Therefore, the data collected on the time axis are resampled in 1 cm intervals in the proceeding direction to apply a consistent function model in the spatial domain. Based on the installed MSR and a buried magnet marker, the effective measurement distance of magnetic signals was found to be within 30 cm, implying that data collection in 1 cm intervals results in a sufficient resolution for signal analysis. When represented on a 3D coordinate system, the data collected by the MSR have a Gaussian distribution, as shown in Figure 5.

#### 3.1.2. Estimation of the Center Point of the Magnetic Field Signal in the Collected 2D Data Space

If the acquired magnetic signals include a peak above the threshold size (the magnetic signal distribution is acquired until the magnetic marker is passed), the position of the peak is estimated on the 2D plane. The magnetic signals have a Gaussian distribution centering on the magnetic marker, and the peak position of the Gaussian distribution is the position of the center of the magnetic marker. The y-axis of the collected data represents the data for the discrete space measured with the magnetic sensors placed at certain intervals, whereas the x-axis represents the data measured at the sampling time and may span between two control cycles (based on a reference control cycle of 50 ms). Because it includes noise signals that distort the signals, the following process is performed to find the peak of the magnetic signal in the discrete space including noise:

1. The position of the maximum [x_max, y_max] is found in the collected 2D data;
2. The top, bottom, left, and right data spaces of a certain size around the position of the maximum are extracted;
3. The sum of each row and the sum of each column in the extracted sample space are calculated;
4. Because only the peak of the signal needs to be determined, the signal is assumed to have a parabolic shape with the maximum point to simplify and reduce the computational process. Then, it is modeled using a quadratic function and the least square method is applied to find the coefficient of the quadratic function. As can be seen in Figure 5, while the overall shape of the acquired magnetic signal has a Gaussian form, it has a quadratic form near the peak. In order to minimize the amount of signal processing data, only a part including the maximum point of the signal is extracted and processed. In addition, since only the x-axis position information with the maximum point of the signal needs to be obtained, the calculation process is simplified by a quadratic function. The validity of this method is confirmed through an actual driving test;
5. To find the maximum point, the position value of a point with a slope of 0 is found by applying the differential of the quadratic function.

**Figure 6:**
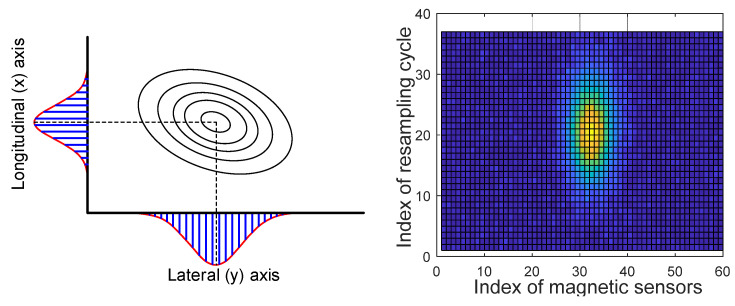
Peak of magnetic field signal in the 2D data space.

**Figure 7:**
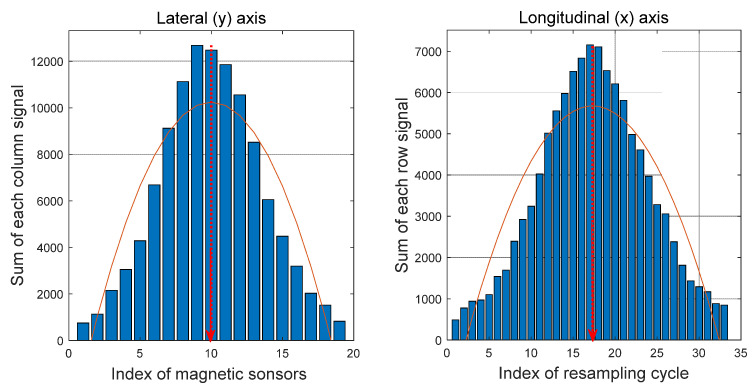
Magnet signal peak for each axis.

As shown in Figure 6, the peak of the signal is created for the x- and the y-axes in each data space, and the same process is performed to check the positions. The results are shown in Figure 7. Due to the limitation of the straight section of the test route, the vehicle was driven with a maximum speed of 25 km/h, and partial signal processing results of the magnetic signal were acquired for driving with a speed of about 15 km/h.

#### 3.1.3. Estimation of the Position of the Center of the Magnetic Marker (Center of the Magnetic Field Signal) in the Coordinates of the Magnetic Sensing Ruler (L_x_, L_y_)

The center position information of the magnet marker found in the magnetic signal collection space is converted into the center position information of the magnet marker in the real-world physical space coordinates using the MSR as the reference axis.

**Figure 8:**
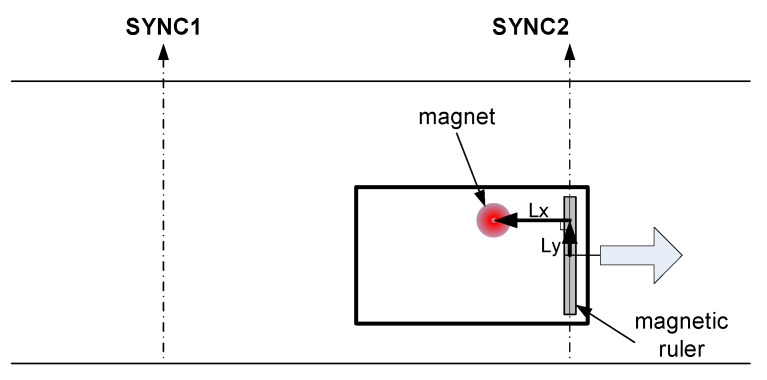
Vehicle local Cartesian coordinate system.

**Figure 9:**
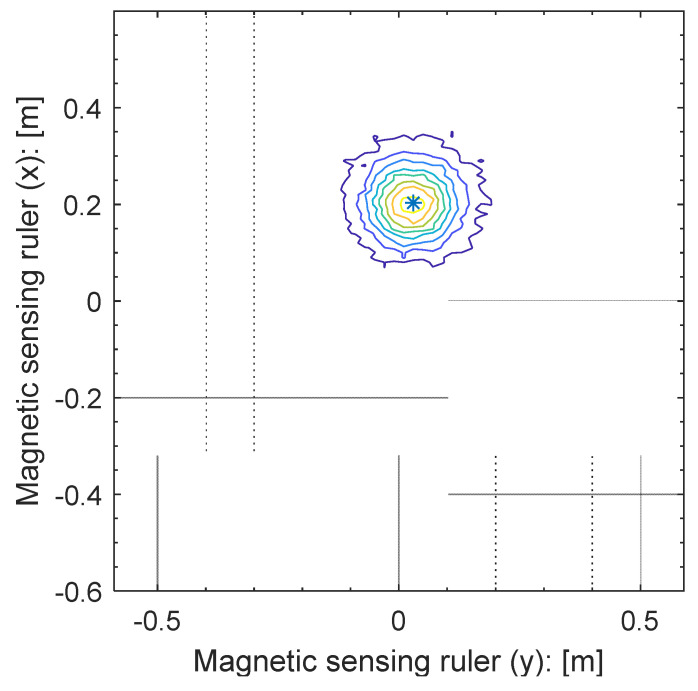
Magnetic marker center position in the sensor coordinate system.

As shown in Figure 8, the detection time of the peak of the magnet signal and the 2D relative distance between the magnet marker and the MSR at that time-point are calculated. Then, the following three pieces of information can be obtained. For this, the distance from the magnet marker detection-based sync to the magnet marker is calculated using the vehicle’s speed and rotational angle at the reference sync and the magnet center detection moving time at the reference sync. Figure 9 shows the magnetic signal contour and the position of the center of the magnet marker in the MSR-based coordinate system. The zero point of the y-axis is the center point on the length of the MSR, and the zero point of the x-axis is the position of the sensor in the control cycle. In Figure 9, the asterisk (*) represents the calculated center point of the magnet marker.

## 4. Localization Based on Magnetic Marker

### 4.1. Position Error Based on the Detected Magnetic Marker

**Figure 10:**
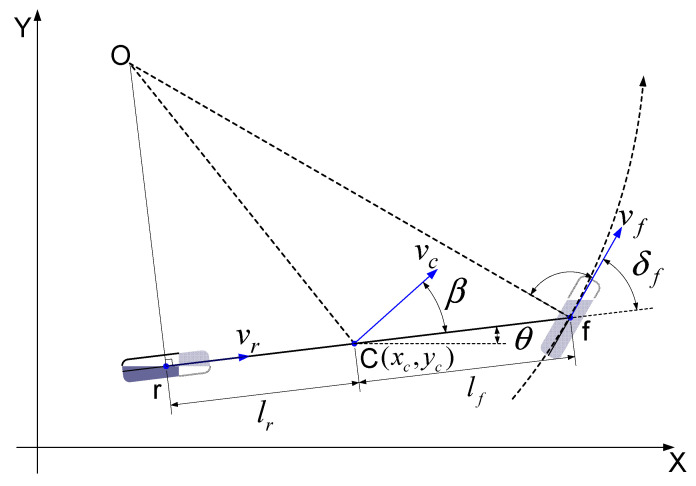
Kinematic model of the vehicle.

If the relative distance (*L_x_*, *L_y_*) of the magnet marker detected based on the MSR coordinate system is determined, the following process is performed to find the absolute position of the vehicle using this information. A bicycle model is applied to simplify the movement of the vehicle, as shown in Figure 10, to calculate the real-time position of the vehicle on the absolute coordinate system. The vehicle’s position is calculated every cycle based on the vehicle’s center axis (C).

Figure 10 depicts the case in which the central axis of the vehicle moves around the center of rotation (o), defining the kinematic motion of the vehicle and associated parameters. In the above figure, *β* is the side slip angle, *δ_f_* is the front wheel steering angle, *l_f_* is the distance from the front axle to the vehicle central axis, *l_r_* is the distance from the rear axle to the central axis, *ν_c_* is the speed at the central axis, and *ν_f_* and *ν_r_* are the speeds of the front and rear wheels, respectively:

(1) $$\begin{array}{l} x_{c}(k+1)=x_{c}(k)+dt\cdot v_{c}\cdot \cos(\theta +\beta )\\ y_{c}(k+1)=y_{c}(k)+dt\cdot v_{c}\cdot \sin(\theta +\beta )\\ \theta (k+1)=\theta (k)+dt\cdot v_{c}\cdot \frac{\cos\beta \cdot \tan\delta_{f}}{l_{f}+l_{r}} \end{array}$$

where $\beta =\tan^{-1}\left(\frac{l_{r}\cdot \tan\delta_{f}}{l_{f}+l_{r}}\right)$ and $v_{c}=\frac{v_{f}\cdot \cos\delta_{f}+v_{r}}{2\cdot \cos\beta }$, and *dt* is the discrete sample time.

To set the vehicle’s initial information, we roughly match the known magnetic marker points on the driving path with the front end of the vehicle. At this time, the vehicle’s initial orientation is set, assuming that the vehicle’s orientation is close to the orientation of magnets by aligning the vehicle as parallel as possible with a straight line between magnets. In addition, the initial approximate vehicle center position (*x_c_, y_c_*) is calculated using the vehicle dimension information and orientation. Once the initial position is set, the position and orientation of the vehicle are calculated for each control cycle using vehicle sensor information (odometers, steering angle sensor, and gyroscope) and vehicle model (Equation (1)) until the magnetic marker is detected. When a magnetic field signal is detected, the position of the vehicle is corrected through the process in Equations (3) to (12). In order to identify the detected magnetic marker from the magnetic marker database, when a magnetic signal of a certain strength or more is measured, the position of the magnetic marker is estimated using Equation (3), and the nearest magnetic marker is obtained from this position as in Equation (4). Moreover, since the position detection error of the magnetic marker is detected within 20 cm throughout the driving test, it is judged that the magnetic object with a position error of 30 cm or more is not a magnetic marker. Even if some magnetic markers could not be detected due to a missing signal or a loss of signal during driving, the position error did not exceed 30 cm even after moving 50 m from the last detected magnetic marker. Therefore, even when magnetic markers are not detected due to some factor, distances greater than 50 m can be easily estimated. For safety reasons, however, the vehicle controller stops the vehicle when there is no magnetic marker detection while moving 15 m after last magnetic marker detection (corresponding to five missing markers in a row) [21]. 

**Figure 11:**
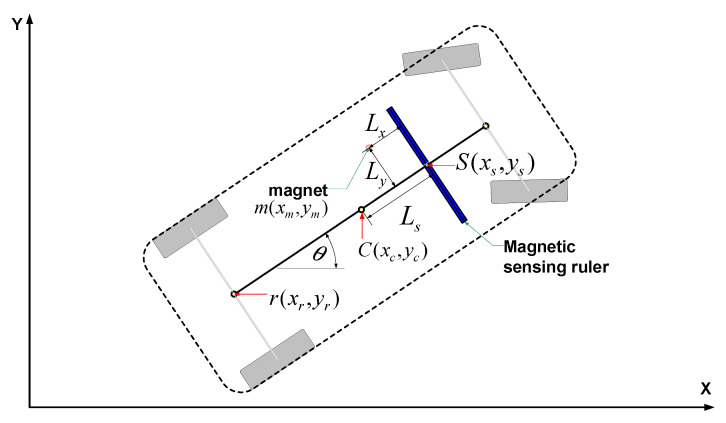
Measurement model with MSR.

Figure 11 shows the relationship between the MSR mounted on the vehicle and the detected magnet marker. The MSR’s position (*x_s_, y_s_*) is calculated based on the vehicle’s center coordinate (*x_c_, y_c_*), as shown in Equation (2):

(2) $$\left[\begin{array}{c} {\hat{x}}_{s}\\ {\hat{y}}_{s} \end{array}\right]=\left[\begin{array}{c} {\hat{x}}_{c}\\ {\hat{y}}_{c} \end{array}\right]+L_{s}\left[\begin{array}{c} \cos\theta \\ \sin\theta \end{array}\right]$$

where *L_s_* is the distance from the center of the vehicle coordinates to the center of the MSR and *θ* is the vehicle’s heading angle.

Then, the absolute position (*x_m_, y_m_*) of the detected magnet marker can be calculated, as shown in Equation (3), using the relative distance information (*L_x_, L_y_*) measured from the MSR’s position (*x_s_, y_s_*) to the detected magnet marker (m).

(3) $$\left[\begin{array}{c} {\hat{x}}_{m}\\ {\hat{y}}_{m} \end{array}\right]=\left[\begin{array}{c} {\hat{x}}_{s}\\ {\hat{y}}_{s} \end{array}\right]+L_{x}\left[\begin{array}{c} \cos\theta \\ \sin\theta \end{array}\right]+L_{y}\left[\begin{array}{c} -\sin\theta \\ \cos\theta \end{array}\right]$$

Based on the index of the closest magnet marker from the estimated position of the magnet marker in the magnet marker database, the magnet marker with the minimum Euclidean distance is found, as shown in Equation (4):

(4) $$(i_{\min},d_{\min})=\min\left(\sqrt{{\left(X_{DB}-{\hat{x}}_{m}\right)}^{2}+{\left(Y_{DB}-{\hat{y}}_{m}\right)}^{2}}\right)$$

where *i*_min_ is the index of the magnet marker that has the minimum distance, *d*_min_ is the minimum distance, and *X_DB_*, *Y_DB_* is the position coordinate array in the magnet marker database.

Therefore, the error of the magnet marker position detected at the estimated magnet marker position becomes the absolute position calculation error of the system, as shown in Equation (5):

(5) $$\left[\begin{array}{c} dx_{e}\\ dy_{e} \end{array}\right]=\left[\begin{array}{c} X_{DB}(i_{\min})\\ Y_{DB}(i_{\min}) \end{array}\right]-\left[\begin{array}{c} {\hat{x}}_{m}\\ {\hat{y}}_{m} \end{array}\right]$$

Furthermore, the error can be calculated and converted into the vehicle coordinate system, as shown in Equation (6):

(6) $$\left[\begin{array}{c} dx_{v}\\ dy_{v} \end{array}\right]=dx_{e}\left[\begin{array}{c} \cos\theta \\ \sin\theta \end{array}\right]+dy_{e}\left[\begin{array}{c} -\sin\theta \\ \cos\theta \end{array}\right]$$

where *dx_v_* is the estimated error in the proceeding direction of the vehicle (mainly due to the vehicle’s wheel radius and slip) and *dy_v_* is the estimated error in the lateral direction of the vehicle (mainly due to the vehicle’s heading error).

The vehicle’s heading error can be calculated using Equation (7).

If the lateral direction error information of the currently detected magnet and the previously detected magnet is used, the vehicle’s heading error can be determined, as shown in Equation (7). The detected magnet marker number is recorded whenever detected, and up to the three of the latest numbers are recorded:

(7) $$d\theta_{e}=sign(dy_{v})\cdot \sin^{-1}\left(dy_{v}/d_{m}\right)$$

where *d_m_* is the Euclidean distance between the two detected magnets.

(8) $$d_{m}=\sqrt{(X_{DB}(i_{\min}(n))-X_{DB}{\left(i_{\min}(n-1)\right)}^{2}+{\left(Y_{DB}(i_{\min}(n))-Y_{DB}(i_{\min}(n-1))\right)}^{2}}$$

To define the sequence number of the detected magnetic marker, *i*_min_*(n)* refers to the current detection and *i*_min_(*n −* 1) refers to the previous detection.

Finally, the absolute position error of the vehicle is determined, as shown in Equation (9):

(9) $$dP={\left[\begin{array}{ccc} dx_{e} & dy_{e} & d\theta_{e} \end{array}\right]}^{T}$$

### 4.2. Time-Division Position Correction

The vehicle’s position error is calculated only when a magnet marker is detected, and it is used to correct the vehicle’s position. Once the position error of the vehicle is determined, it is corrected by dividing it within the maximum installation interval (3 m) until the next magnet marker is detected. If the position error is large and corrected when a magnet is detected, a discontinuity of the position information may occur, causing control shock in the steering control that uses this error, which may consequently strain the steering system or reduce the ride comfort of the passengers. Therefore, a time-division correction method is proposed to prevent this.

#### 4.2.1. Determination of the Number of Divisions

The absolute position error calculated by detecting a magnet marker is a constant until the next magnet marker is detected. Therefore, the number of divisions for this value is determined according to the vehicle’s speed, as shown in Equation (10), to determine the method of dividing and correcting the error in every control cycle until the next magnet marker is detected. In other words, the lower the speed, the higher the number of divisions. This method was determined to complete the determined correction between the magnet markers:

(10) $$Count_{mk}=\frac{Dis}{\left(\left|v_{c}(k)\right|\cdot dt+eps\right)}$$

where *Dis* is the maximum interval (3 m) of magnet markers, *v_c_*(*k*) is the vehicle speed, *dt* is the discrete sample time of control cycles, and *eps* is a small value utilized to prevent the denominator from becoming zero when the speed is 0.

Equation (11) shows the condition equation for division correction:

(11) $$\begin{array}{l} \mathrm{if}\;Count_{up}<Count_{mk}\\ \;dp1=dP/Count_{mk};\\ \mathrm{else}\\ \;dp1={\left[0\;0\;0\right]}^{\prime };\\ \mathrm{end}\\ \mathrm{if}\;sum(abs(dP)<dP1\_sum)\cup (abs(v_{c}(k))<0.01)\\ \;dp1={\left[0\;0\;0\right]}^{\prime };\\ \mathrm{else}\\ \;Count_{up}=Count_{up}+1;\\ \;dP1\_sum=dP1\_sum+abs(dp1);\\ \mathrm{end}\\ \mathrm{if}\;Count_{up}\;>\;Count_{mk}\\ \;Count_{up}=Count_{mk};\\ \mathrm{end} \end{array}$$

#### 4.2.2. Position Correction

When the correction amount is determined, as shown in Equation (9), after the magnet marker is detected, the division is determined according to Equations (10) and (11). Then, Equation (12) is additionally applied to the position calculated at each cycle by Equation (1) to correct the vehicle’s absolute position:

(12) $$\left[\begin{array}{c} {\hat{x}}_{c}\\ \begin{array}{l} {\hat{y}}_{c}\\ \theta \end{array} \end{array}\right]=\left[\begin{array}{c} {\hat{x}}_{c}\\ \begin{array}{l} {\hat{y}}_{c}\\ \theta \end{array} \end{array}\right]+dp1$$

where *dp*1 is the position correction amount determined after detecting the magnet marker.

## 5. Results and Discussion

**Figure 12:**
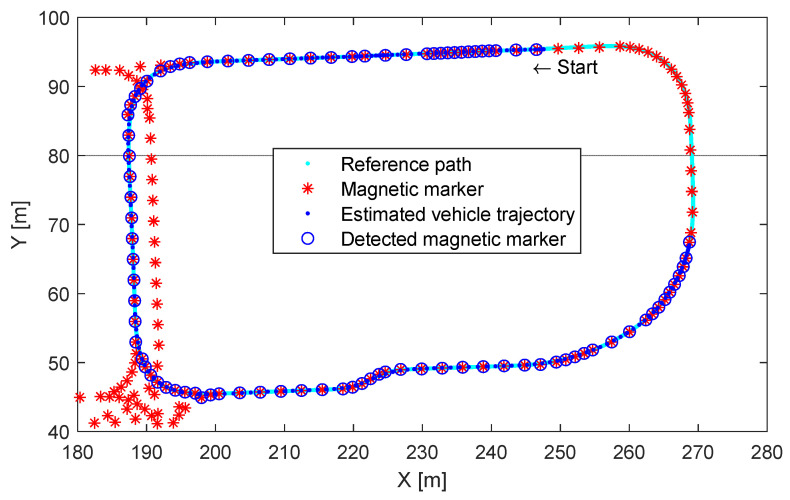
Magnetic-marker-based position estimation and guidance control test results.

We conducted a driving test to demonstrate the performance of the position correction algorithm proposed in this study. The position estimation and the driving path guidance control were performed based on magnet marker detections, and as shown in Figure 12, 112 magnet markers were buried in the driving track. The vehicle was driven in a loop with a length of approximately 238 m. In Figure 12, the light blue dots represent the reference path and the dark blue dots represent the calculated vehicle trajectory. Furthermore, the asterisk (*) indicates the positions of the magnet markers buried in the road and the unfilled blue circle (“o”) indicates the magnet marker positions detected while driving.

**Figure 13:**
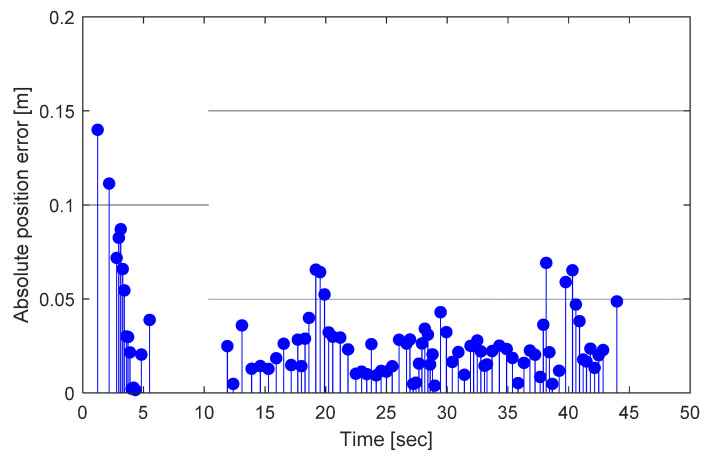
Absolute position error based on the magnetic marker.

The position estimation errors while driving are based on the magnet markers. Figure 13 shows the error in the Euclidean distance between the magnet marker position in the database and the magnet marker position estimated when the magnet marker is detected. Starting with the initial position error based on the approximate initial vehicle position information, the error decreases to below 10 cm as the magnet marker detection is repeated. The mean position detection error of the 91 detected magnet markers was 2.86 cm.

**Figure 14:**
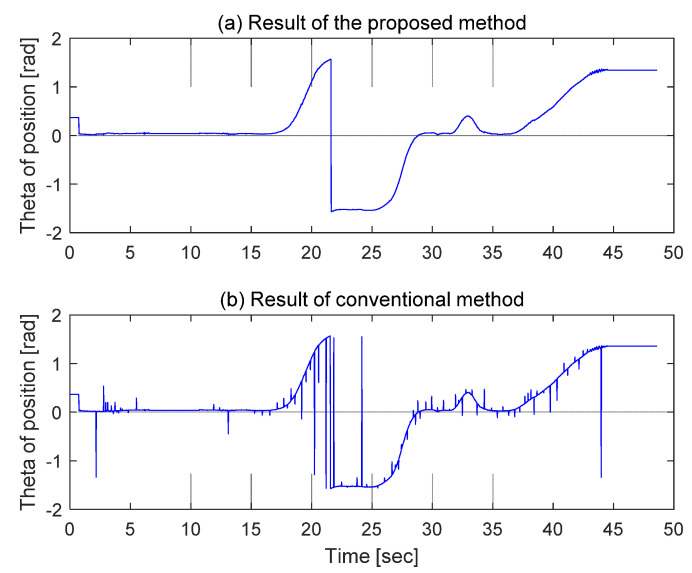
Comparison of continuity [tan(*d_y_*/*d_x_*)] between estimated position points.

Because it was difficult to verify the continuity between the data points from the diagram of the estimated position information, we assessed the continuity between the points using the slopes and the estimated position points to verify the effectiveness and validity of the proposed position correction method. If the calculated current position coordinate (*x_k_,y_k_*) and the previous position coordinate (*x_k*−1*_,y_k*−1*_*) are available, the slope between position points (tan(*y_k*−1*_* − *y_k_*/*x_k*−1*_* − *x_k_*)) can be applied. Figure 14a shows the slopes calculated between the position coordinates as estimated by the proposed method, while Figure 14b shows the slopes calculated between the position coordinates by applying the conventional method, with irregular pulse values in many sections of the graph. In these sections, the discontinuity of the slopes between the position points is shown because the position error is reflected directly whenever a magnet marker is detected. Alternatively, for the results of the proposed method, there is a clearly improved continuity of the slopes, as shown in Figure 14a.

**Figure 15:**
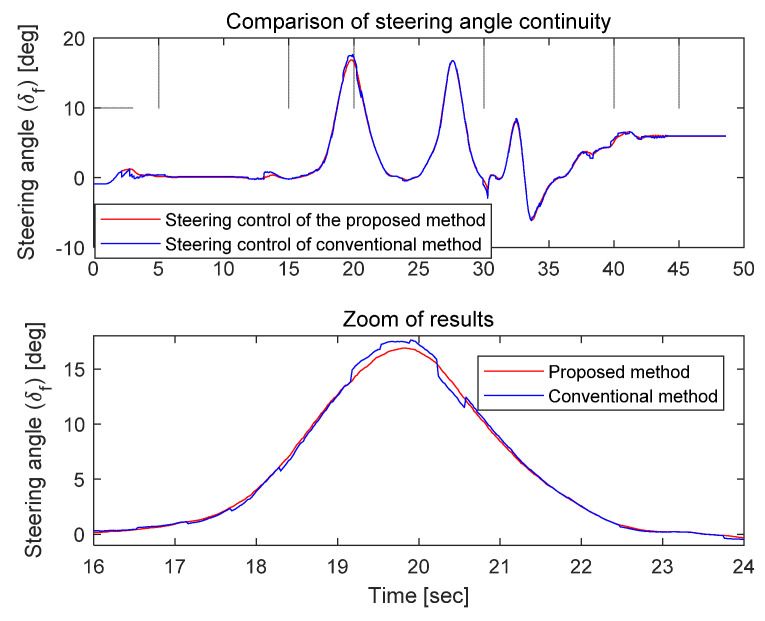
Comparison of steering angle command of path-following controller.

Figure 15 shows the steering angle command results when the same position control was performed, based on the position coordinates calculated derived from the conventional position correction method and the proposed position correction method. As shown in Figure 15, the discontinuity of the position information affected the steering angle calculation, causing discontinuous command values of the steering angle in the conventional method. In contrast, smoothly connected steering angle commands were generated by the proposed method. This can have significant effects on the vehicle’s driving stability and the passengers’ ride comfort with respect to controlling the vehicle. As an additional example, if a common method such as a Kalman filter is used and the filter coefficient is adjusted significantly to mitigate the discontinuity of the position information, the continuity of the position information will improve, although a delay will occur in the position information. In this case, the estimated driving trajectory will be larger compared to the actual driving position at a curve in the road. Furthermore, the error may increase as the vehicle speed increases.

## 6. Conclusions

In this study, we estimated vehicle positions in real-time by detecting magnet markers and proposed novel position estimation and error correction methods. 

Solutions to two main problems in magnetic marker positioning system performance were proposed as follows:

- The speed of sensor utilization was improved through separate processing of the vehicle control cycle and magnet signal detection cycle;
- The continuity of position information was improved using the time-division position correction method.

In automatic driving control systems, the precision and continuity of the real-time position of the vehicle are important elements with respect to the vehicle position control and designed driving path. In particular, we examined the discontinuity of position information, which may occur in land marker methods, such as with the use of magnet markers, and verified its effects. Accordingly, position estimation and error correction methods were then proposed to prevent such discontinuity.

A discontinuity of the position information will occur when magnet markers are detected while driving if the position is only corrected at each detected time-point. Therefore, the calculated position error value is corrected by dividing it while driving according to the proposed procedure. If the error correction is performed by dividing the moving space until the next magnet is detected, the correction value can be used without loss while ensuring there is no sharp discontinuity when the position is corrected.

To verify the validity of the proposed method, we demonstrated the problems that occur in the conventional method of estimating and correcting positions using the test data and compared the performance to that of the proposed method. Based on this comparison, the superiority of the proposed method was confirmed.

## Data Availability

The data presented in this study are available on request from the corresponding author.
